# Potential risks of treating bacterial infections with a combination of β-lactam and aminoglycoside antibiotics: A systematic quantification of antibiotic interactions in *E. coli* blood stream infection isolates

**DOI:** 10.1016/j.ebiom.2022.103979

**Published:** 2022-04-01

**Authors:** Nikos Fatsis-Kavalopoulos, Lex Roelofs, Dan I. Andersson

**Affiliations:** Uppsala University, Dept. of Medical Biochemistry and Microbiology, Uppsala, Sweden

**Keywords:** FICi, Synergy, Antagonism, Additivity, Antibiotics, FICi, Fractional Inhibitory Concentration index

## Abstract

**Background:**

Treatment of Blood Stream Infections (BSIs) with a combination of a β-lactam and an aminoglycoside antibiotic is widely used in intensive care units (ICUs) around the world. However, no studies have systematically examined how these drugs interact and potentially influence the antimicrobial efficacy of the overall treatment.

**Methods:**

We collected 500 E. *coli* isolates from the Uppsala University hospital that were isolated from blood of patients with suspicion of infection. Of those we tested the efficacy of combinations of 2 common β-lactam antibiotics (Ampicillin and Cefotaxime) combined with 2 common aminoglycosides (Gentamicin and Tobramycin) on 254 isolates. The efficacy of all 4 pairwise combinations in inhibiting bacterial growth was then examined on all susceptible strains. That was done by quantifying the Fractional Inhibitory index (FICi), a robust metric for antibiotic combinatorial behaviour, of all possible treatments on every strain. When non additive interactions were identified, results of the original screen were verified with time kill assays. Finally, combination behaviours were analysed for potential cross correlations.

**Findings:**

Out of the 4 antibiotic combinations screened none exhibited synergistic effects on any of the 254 strains. On the contrary all 4 exhibited important antagonistic effects on several isolates. Specifically, the combinations of AMP-GEN and CTX-GEN were antagonistic in 1.97% and 1.18% of strains respectively. Similarly, the combinations of AMP-TOB were antagonistic on 0.78% of all strains. PCA analysis revealed that an important factor on the responses to the combination treatments was the choice of a specific aminoglycoside over another. Subsequent cross correlation analysis revealed that the interaction profiles of combinations including the same aminoglycoside are significantly correlated (Spearman's cross correlation test p<0.001).

**Interpretation:**

The findings of this study elucidate potential risks of the common combination treatment for blood stream infections. They also demonstrate, previously unquantified metrics on how antibiotics in combination therapies are not interchangeable with others of the same class. Finally, they reiterate the need for case-by-case testing of antibiotic interactions in a clinical setting.

**Funding:**

This work was funded by grants to DIA from the Swedish Research Council, the Wallenberg foundation and the Swedish Strategic Research Foundation.

**Research in context**

**Evidence before this study**

The combination of β-lactam/aminoglycoside antibiotics has been widely used for the treatment of Blood Stream infections (BSIs). However, reports on the merits of this empirical treatment are conflicting. Several studies report no benefit of the drug combination compared to β-lactam monotherapy, with some reporting even greater efficacy of β-lactam monotherapy altogether. In contrast, other studies and metanalyses report an association between the use of this combination and improved clinical outcome. Conflicting are also the reports that study antibiotic resistance prevention, with some stating that combination therapy better protects against the rise of resistant infections and others finding no improved effect. There have been sporadic attempts to systematically quantify the effects of this drug combination, but the number of clinical isolates tested is insufficient to discern large scale trends.

**Added value of this study**

In this study, we set out to investigate the combinatorial efficacy of the most common antibiotic combination used in the treatment of Blood stream infections. We chose to test 4 such combinations: Gentamicin (GEN) or Tobramycin (TOB) combined with Ampicillin (AMP) or Cefotaxime (CTX). To this end, we performed the largest to date systematic quantification of antibiotic interactions in clinical isolates. Our study encompasses 254 patient samples of E. *coli* strains isolated from blood of patients with suspicion of a BSI. For most strains we detected an additive interaction between the antibiotics used in combination. Alarmingly in a fraction of our isolates we discovered, previously overlooked, significant levels of antagonism between some antibiotics. Specifically, the combination of AMP-GEN was antagonistic in approximately 2% of strains followed by the combinations of CTX-GEN and AMP-TOB that were antagonistic in approximately 1% of strains. A principle-component analysis on the interaction profile of the strains tested revealed that the interaction of the 2 antibiotics in these combinations largely depends on the choice of the aminoglycoside used.

**Implications of all the available evidence**

Our findings raise concerns in the common empirical practice of combination therapy for treating BSIs. In combination therapy schemes, synergy between the antibiotics used is preferred and antagonism between them is to be avoided. Although the vast majority of strains exhibited an additive (neither positive or negative) interaction between the antibiotics used, a significant number of them demonstrated antagonism with one isolate even exhibiting a suppressive effect in one combination. Not only is it concerning to detect antagonistic behaviours between antibiotics in clinical strains, but perhaps of more important note is that not a single case of antibiotic synergy was found. Finally, our data implies that the common practice of using antibiotics within the same class interchangeably might not be ideal, since behaviours in combination therapies seem to change depending on which antibiotics of the same class are used.

Alt-text: Unlabelled box

## Introduction

A single antibiotic is often sufficient to treat the majority of acute infections caused by susceptible bacteria. However, depending on the bacterial species and the site of infection, some infections are treated with combinations of multiple antibiotics.^1^^,^^2^ One motivation for using drug combinations is to reduce the risk of *de novo* evolution of resistance, as is the case in tuberculosis (TB) infections that are treated with several different antibiotics.^3^ Similarly, a combination of drugs may also reduce the impact of pre-existing resistance on treatment outcomes, as observed in the widely used combination of a β-lactam antibiotic and a β-lactamase inhibitor.^4^ Finally, antibiotic combinations are very commonly used for life-threatening infections, when rapid treatment is necessary and the causative agent is unknown. Such empirical treatments are implemented with the reasoning that multiple antimicrobials provide broader coverage in comparison to that of a single antibiotic.^5^^,^^6^

Under that premise, empirical antibiotic treatments find extensive use in severe cases of Blood Stream Infections (BSIs), like sepsis and septic shock.^7^ They are preferred mainly to broaden the spectrum of antimicrobial activity^7^^,^^8^ but also to achieve a potential synergistic effect^9^, ^10^, ^11^ between multiple antibiotics. Even milder cases are often treated empirically at first with a broad spectrum combination of antibiotics.^8^^,^^12^

BSIs in general pose a therapeutic challenge, which is further magnified by the rise of antimicrobial resistance.^13^^,^^14^ Approximately 28% of all patients admitted to intensive care units suffer from some form of bacteremia or sepsis^15^^,^^16^, and an additional 18% acquire BSIs during hospitalization.^16^ These infections have mortality rates as high as 30% in hospitals in high income countries.^17^

Of all potential pathogens *E. coli* is the most commonly responsible for BSIs.^18^, ^19^, ^20^ It accounts for approximately 27% of all bacteremia cases in adults worldwide, with some studies reporting as high as 57%.^21^ It is furthermore, the pathogen with the fastest growing number of cases per year.^18^ A variety of *E. coli* primary infections can result in bacteremia or sepsis including surgical site infections^22^^,^^23^, ventilator-associated pneumonia^24^, ^25^, ^26^, abdominal and pelvic infections^27^^,^^28^ and urinary tract infections.^29^, ^30^, ^31^

On a suspected E. *coli* blood stream infection, the first line treatment guidelines in Europe the US and specifically in Sweden, dictate as a first course of action the use of a broad spectrum combination of an aminoglycoside with a β-lactam.^12^^,^^32^, ^33^, ^34^, ^35^, ^36^

Although β-lactam/aminoglycoside combinations are extensively used, there is an important parameter of this combination therapy that is often left unchecked. That is the effect the interactions between the drugs have on the overall treatment efficacy. Ideally the drugs used in the same treatment should synergize to a combined inhibition greater than the sum of each individual effect (antibiotic synergy). In some cases, however the drugs antagonize each other to a combined inhibition that is less than the sum of the individual effects (antibiotic antagonism). The differences between synergistic and antagonistic cases are important to take into account, especially since reports on whether or not β-lactam/aminoglycoside combinations result in better clinical outcome are conflicting.^7^

In this study, we aimed to characterize the antibiotic interaction profiles *in vitro* of one of the most commonly prescribed antibiotic combinations consisting of a β-lactam combined with an aminoglycoside for treatment of BSIs. We set out to systematically quantify the interactions of the β-lactam/aminoglycoside combination, against an extensive collection of BSI E. *coli* clinical pathogens. We quantified *in vitro* the combined antibacterial effects of two commonly used aminoglycosides, Gentamycin and Tobramycin (GEN and TOB), and two β-lactams, Ampicillin and Cefotaxime (AMP and CTX), for 254 *E. coli* BSI clinical strains. We then characterized the interaction of every antibiotic combination as synergistic, antagonistic or additive. A majority of the strains showed additive interactions, no strains demonstrated a synergistic response and, unexpectedly, a substantial fraction of the strains exhibited an antagonistic response. These findings demonstrate the potential risks of β-lactam/ aminoglycoside combinations and the need for isolate-specific testing to identify cases of antagonism in which the efficacy of the antibiotic combination might be reduced.

## Methods

### Strains, growth conditions and culture media

All strains (Supplementary file 1) were isolated from purified clinical samples and frozen as purified single clones in 10% DMSO in LB solution. Clinical strains were acquired from Uppsala University Hospital, and were isolated from patients with positive blood cultures admitted in the hospitals ICU. The demographic of the hospital spans from urban to suburban and rural. the strain collection is composed of strains isolated from positive blood cultures of suspected bacteremia cases (from 2014-2017 to provide a sufficiently large strain collection), as part of standard care (i.e no ethical permit was needed). The collection was provided from Uppsala University Hospital and screened and no power calculations were performed as the entire available strain population was screened. For all subsequent experiment strains were streaked from frozen stock on Mueller-Hinton agar plates (Difco^TM^, product number: 225230) and incubated overnight at 37°C. A single colony was picked from every plate and incubated in 1 ml Mueller-Hinton broth (Difco^TM^, product number: 275730) overnight and incubated at 37°C, 195 rpm orbital shaking.

### Antibiotic stocks

Antibiotic stocks were prepared from powder stock, in sterile nuclease-free water (Sigma Life Sciences, product number: W4502-1L) on the same day of use according to manufacturer's guidelines. AMP, CTX, GEN and TOB were all ordered from Sigma-Aldrich (product number: 102240069, C7039-1G, G1914-5G, T1783-500MG respectively).

### Testing of susceptibility and interactions

Minimum Inhibitory Concentrations (MICs) were calculated using the broth microdilution method. A two-fold serial dilution of AMP, GEN, CTX or TOB was prepared in 96-well microtiter plates in triplicates. Overnight cultures prepared in broth as described above were inoculated in a final volume of 180 μL in each well (containing approximately 5 × 10^5^ cells). Plates were incubated overnight at 37°C with no shaking. Before measuring growth on the plates, the medium in all wells was resuspended. Inhibition of growth was measured by means of optical density measurements at 540 nm using a Multiscan FC Type 357 (Thermo Fisher Scientific). MICs were called on the well containing the lowest concentration of antibiotics sharing the same OD as control wells with no bacteria. Interaction testing was performed using CombiANT on susceptible strains, an antibiotic diffusion based method that quantifies antibiotic interactions in bacterial cultures as described in previous work.^37^ Susceptibility was defined according to EUCAST guidelines (supplementary data 2 on proportion of strains found resistant). Each isolate was tested for all possible aminoglycoside + β-lactam combinations (i.e. Ampicillin in combination with Tobramycin or Gentamycin and Cefotaxime in combination with Tobramycin or Gentamycin) in triplicate, then FICi was defined as the average between the 3 values. The Fractional Inhibitory Concentration Index (FICi), was used as a measurement of the interaction between two antibiotics ^38^. According to accepted clinical thresholds for FICi values^39^, ^40^, ^41^, ^42^, interactions scored with a FICi >4 = antagonistic, FICi <0.5 = synergistic, 1<FICi<4, >1 = additive to antagonistic and 0.5<FICi <1 = additive to synergistic (supplementary data 1 on interaction indices for all strains).

### Time-kill assays

Triplicate overnight cultures were grown from three separate single colonies in 2 ml LB. Overnight cultures were diluted in 4 different 10 ml tubes containing 2 ml Mueller-Hinton broth (MHB)to an approximate of 8 × 10^6^ cells per tube. Antibiotics were then added to each of the four tubes as follows: Tube 1: antibiotic A, Tube 2: antibiotic B, Tube 3: antibiotic A + antibiotic B, Tube 4: no antibiotic control. The amount of antibiotics added was calculated to be at MIC levels for every strain in all conditions. The tubes were incubated together at 37°C with orbital shaking (200 rpm). At every time point (0h, 2h, 4h, 8h and 24h) 20 μL of bacterial suspension was sampled from each tube. The sample was then diluted and plated on agar plates in 5 μL droplets according to Miles and Misra spotting technique.^43^ Plates were incubated overnight at 37°C and following incubation, colony forming units (CFU) were counted. Stability of counts was confirmed by extended incubation of up to 48 hours. Survival was calculated as the amount of CFU of a condition compared to the initial CFU of that condition in the 0h time point. The theoretical additive effect of the combination was calculated from multiplying the survival rates of the 2 antibiotics, according to the Bliss independence model of antibiotic interactions.^44^ According to Bliss independence if two compounds are acting independently then their combined effect to bacterial survival is the multiplicative effect of each individual drug's inhibition.

### Statistical and data analysis

Statistical analysis was performed in Graphpad Prism 9 version 9.1.1 for Windows. Principal component analysis was performed on a standardized scale. The input was the FIC indices of all 4 interactions for every strain. Principal components were picked by performing a parallel Monte Carlo analysis on randomized data.^45^ Principal components with eigenvalues greater than the randomized dataset at the 95% level were selected. The correlation Spearman correlation matrix shows the correlation coefficient the FIC index of one antibiotic combination and the FIC index of the other 3 combinations, on this strain collection.

### Role of the funding source

The funding bodies had no say in data analysis, collection, interpretation, or decision to publish.

## Results

The study material consisted of 500 isolates from patients with *E. coli* blood stream infections admitted to Uppsala University Hospital on suspicion of infection between January 1^st^ 2014 and December 31^st^ 2017. Two β-lactams, AMP and CTX, were screened for interactions with two commonly used aminoglycosides, GEN and TOB. Out of the 500 strains screened, 254 (50.8%) were identified as susceptible to all four antibiotics and analysed further.

**Figure 1:**
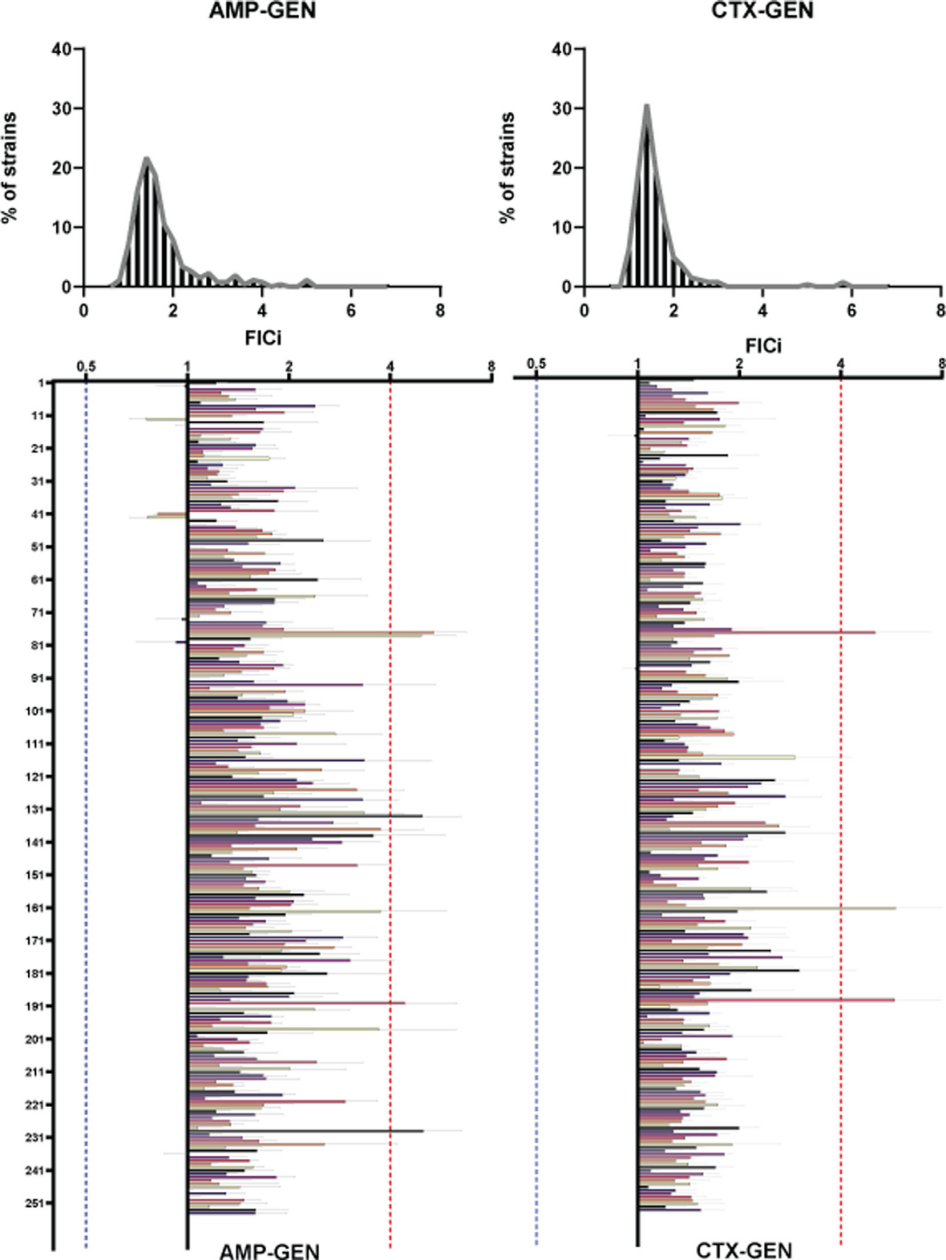
Interaction profiles of the Combinations of AMP (left) and CTX (right) with GEN on all strains. The distribution of strains across FICi values is represented on the top followed with an exhaustive representation of all strains (bottom). Y axis denotes a sequential number for every strain, the corresponding ID number can be found in supplementary file 1. X axis denotes FICi values. The X axis is transformed to a log2 representation, FICi=1 is the zero level of the axis, values < 1 are represented as bars spanning from the zero level to the left, values >1 are represented as bars spanning from the zero level to the right. Every strain is represented with its mean FICi value (n=3) and standard deviation. The red dotted lines represent the clinical level for antagonism (FICi=4), the blue dotted lines represent the clinical levels for synergy (FIC=0.5).

In the combinations of GEN with either β-lactam, no synergy between the antibiotics was found in any of the strains (Figure 1). Both combinations had additive to antagonistic effects in the vast majority of strains, with the antibiotic pairs having additive to synergistic responses in very few strains (Table 1). More concerning was the finding that 1.97% of strains (strainID: DA63190, DA63824, DA63192, DA63980, and DA 63614) in the combination of GEN with AMP and 1.18% of strains (strainID: DA63192, DA63824, and DA6370) in the combination of GEN with CTX, exhibited significant levels of antagonism. Furthermore, two strains (strainID: DA63192 and DA63824) (0.78%) were shown to have an antagonistic interaction in both combinations.

**Table 1:** The interactions of β-lactams with Gentamicin. The percentages out of 254 strains tested that fall under the different kinds of interactions. AMP-GEN is the combination of Ampicillin with Gentamicin and CTX-GEN is the combination of Cefotaxime with Gentamicin.

Table 1	AMP-GEN	CTX-GEN
Synergistic (FICi<0.5)	0%	0%
Additive to Synergistic (0.5<FICi<1)	3.14%	0.78%
Additive to Antagonistic (1<FICi<4)	95%	98.03%
Antagonistic (FICi>4)	1.97%	1.18%
Antagonistic on both β-lactams	0.78%	

**Figure 2:**
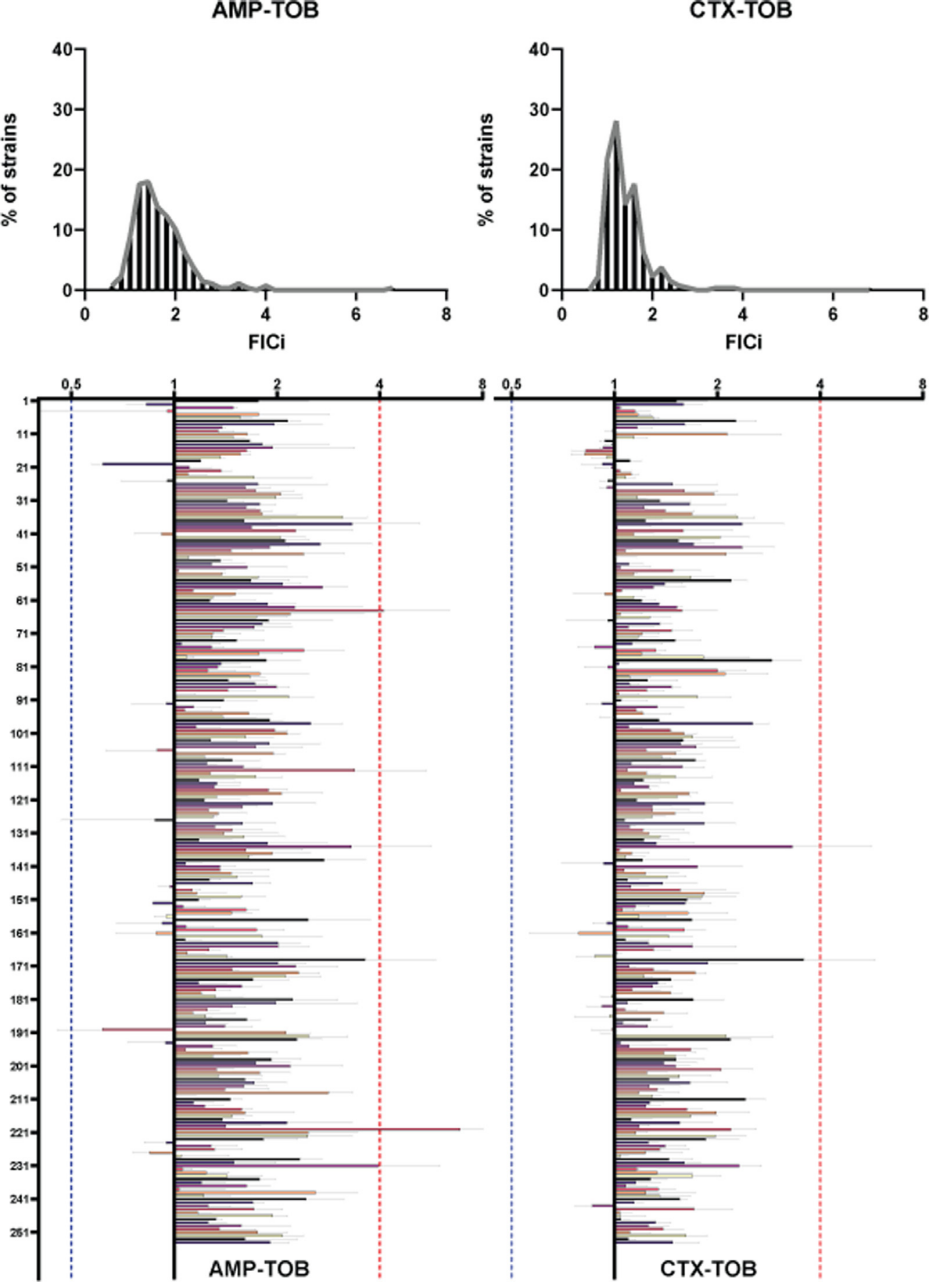
Interaction profiles of the combinations of AMP (left) and CTX (right) with TOB on all strains. The distribution of strains across FICi values is represented on the top followed with an exhaustive representation of all strains (bottom). The Y axis denotes a sequential number for every strain, the corresponding ID number can be found in supplementary file 1. X axis denotes FICi values. The X axis is transformed to a log2 representation, FICi=1 is the zero level of the axis, values < 1 are represented as bars spanning from the zero level to the left, values >1 are represented as bars spanning from the zero level to the right. Every strain is represented with its average FICi value (n=3) and standard deviation. The red dotted lines represent the clinical level for antagonism (FICi=4), the blue dotted lines represent the clinical levels for synergy (FIC=0.5).

In the combinations with TOB, no antibiotic pairs were shown to have a synergistic effect against any of the strains tested (Figure 2). Similar to the combinations using GEN, additive to antagonistic effects were found in the majority of the strains (Table 2). Additionally, the TOB combinations were found to be additive to synergistic in more strains in comparison to the combinations using GEN (6.69% vs 3.14% for AMP and 10.62% vs 0.78% for CTX). Finally, clinical levels of antagonism between AMP and TOB were detected in 0.78% of strains (strains: DA63192, DA63980), and both antibiotic combinations containing TOB were not found to be antagonistic in any strain.

**Table 2:** The interactions of β-lactams with Tobramycin. The percentages out of 254 strains tested that fall under the different kinds of interactions. AMP-TOB is the combination of Ampicillin with Tobramycin and CTX-TOB is the combination of Cefotaxime with Tobramycin.

Table 2	AMP-TOB	CTX-TOB
Synergistic (FIC<0.5)	0%	0%
Additive to Synergistic (0.5<FIC<1)	6.69%	10.62%
Additive to Antagonistic (1<FIC<4)	92.51%	89.37%
Antagonistic (FIC>4)	0.78%	0.00%
Antagonistic on both β-lactams	0%	

**Figure 3:**
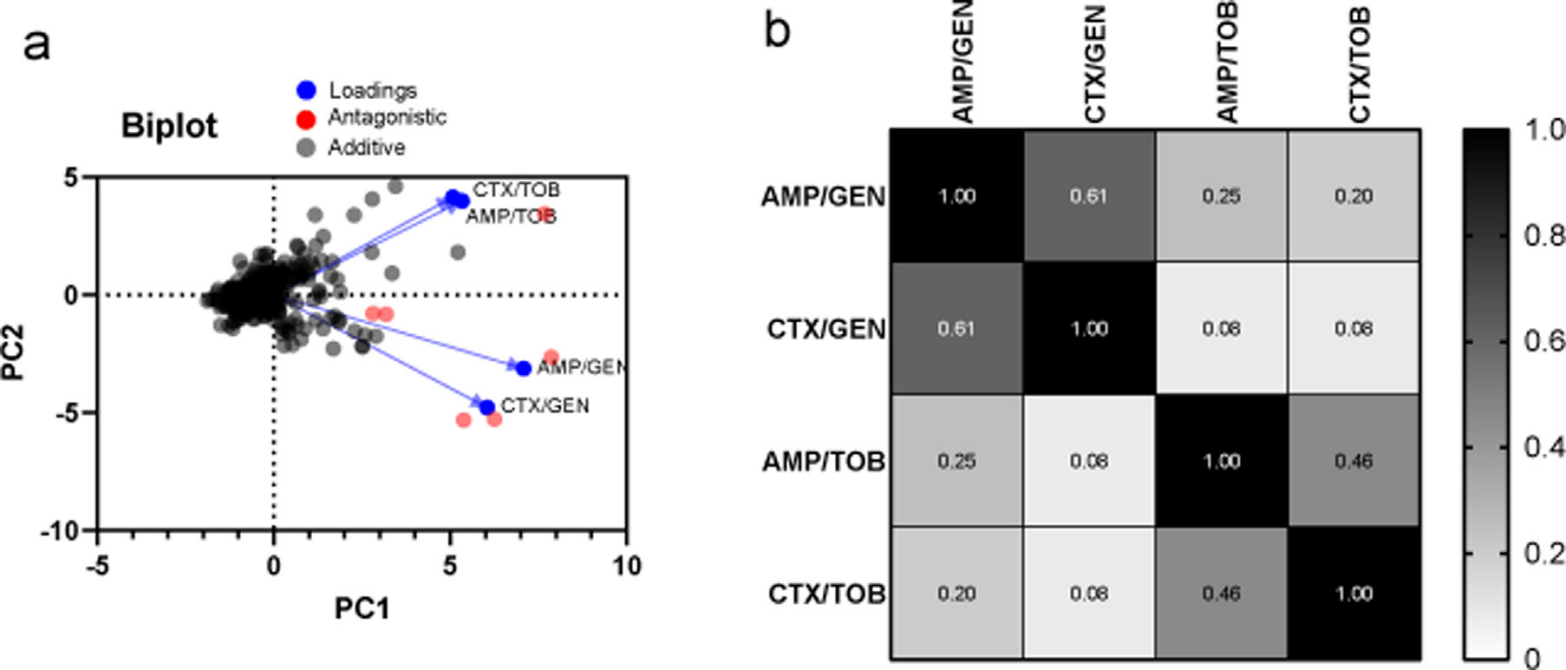
Correlation analysis of the interaction profiles between all antibiotic pairs. a) The Principal Component Analysis Biplot, dots denote individual PC scores for every strain and antibiotic pair. Grey dots represent cases of additive interactions and red dots represent cases of antagonistic interactions. b) Spearman cross-correlation matrix. The value on every square is the Spearman correlation coefficient between the FICi of the combination on that row with the FICi of the combination on that column of the matrix. Colour of the square is a representation of the numerical value with 0 being depicted as white and 1 being depicted as black. CI for all correlation coefficients are as follows: AMP/GEN-CTX/GEN 0.56 to 0.66, AMP/GEN-AMP/TOB 0.01 to 0.28, AMP/GEN-CTX/TOB 0.03 to0.27, CTX/GEN-AMP/TOB -0.14 to 0.10, CTX/GEN-CTX/TOB -0.02 to 0.2 and AMP/TOB-CTX/TOB 0.3 to 0.50

As is evident from Tables 1 and 2, the distribution of strains across the different interaction types seems to be different for every β-lactam + aminoglycoside combination. However, some common trends could be detected; for example, the combinations were overwhelmingly additive to antagonistic and never synergistic. To examine these trends, we subsequently analysed how the four antibiotic combinations correlate between them, in the context of the interaction profiles they exhibit in this set of strains (quantified by FICi values). We performed a principle-component-analysis that yielded two principle-components that account for 78% of all variance between the data (Supplementary Figure 1). As seen in Figure 3a, data points cluster around the (0,0) point of the biplot, indicating a small effect of the combination used on the outcome of the interaction profile. However, the antagonistic outliers are more spread, indicating that for strains exhibiting an antagonistic interaction profile, the antagonism is more dependent on the specific combination used.

The loadings for AMP-TOB and CTX-TOB are clustered together (Supplementary Figure 1), indicating a possible correlation between the interaction profile of these two antibiotic combinations. The loadings for the interaction profiles of AMP-GEN and CTX-GEN suggested a similar correlation. These observations were verified by examining the cross correlations between the interaction profiles of the four antibiotic combinations. As illustrated in Figure 3b, the Spearman correlation coefficients for the interaction profiles observed in the AMP-GEN and CTX-GEN combinations are substantial (61%) and significant (p<< 0.001). Similarly, the interaction profiles observed in the CTX-TOB and AMP-TOB combinations are also correlated (46%) significantly (p<<0.001). No other substantial correlations were present.

**Figure 4:**
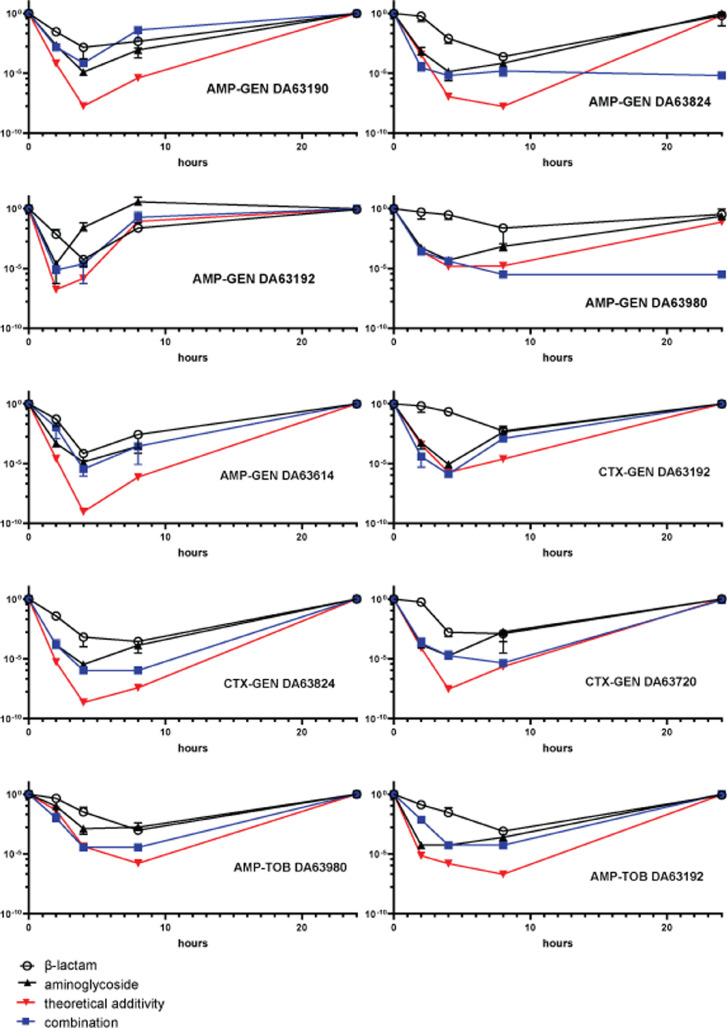
Time Kill assays on selected strains with antagonistic interactions. In every panel the antibiotic pair and the strain ID are displayed on the bottom right. Time points used are 0h, 2h, 8h and 24h. The Y axis denotes survival compared to the initial amounts of cells inoculated. The two black lines represent the individual killing effects of the antibiotics. The black line with circles is the time kill effect of the β-lactam, the line with triangles is the time kill effect of the aminoglycoside. The blue line is the time kill curve of the combination and the red line is the theoretical additive effect the two antibiotics would have based on their individual time kill curves. Increased survival of the combination of antibiotics compared to the theoretical additive indicates antagonism, decreased survival compared to theoretical additive indicates synergy. Representative additive interaction time kills can be found in Supplementary figure 2 for comparison.

We continued by further investigating the cases where an antibiotic combination proved to be antagonistic against a strain. We performed time kill assays to confirm by a different assay the effect of the combination compared to each drug individually (Figure 4). In the cases of antagonistic interaction between AMP and GEN, the combined effect of the antibiotics had higher survival rates of the microbial population compared to theoretical effects of an additive combination. Overall, in most instances, this antibiotic combination had a smaller effect on bacterial survival than the theoretical additive model. One exception was strain DA63980, for which the 2- and 4-hour time points had only marginally larger bacterial survival than the theoretical model, only for the combinatory effect to become smaller in subsequent time points. In 3 of the 5 cases, the antibiotic did not prevent resurgence, with the bacterial population recovering after 24 hours even when treated with both antibiotics. Of special note is the case of strain DA63190, where the combination of AMP with GEN had less effect on bacterial survival than the individual treatment with GEN at all time points. At the 8h time point, the combination even had less of an effect than either of the two antibiotics individually, indicating a suppressive effect.

In the strains where CTX-GEN had an antagonistic profile, the effects of the combination on survival were again overwhelmingly smaller than the theoretical additive interaction. One strain (DA63192) exhibited an additive profile between the two antibiotics until the 8h time point, where the combination of the antibiotics had a substantially smaller effect than the theoretical additive. In all cases the antibiotic combination failed to prevent resurgence, and the populations recovered after 24 hours. Similar to the two previous combinations, strains identified as having an antagonistic interaction with the AMP-TOB combination exhibited overwhelmingly higher survival when treated with the combination than the theoretical additive model predicted. In both cases, the populations recovered after 24 h.

## Discussion

Even though antibiotic combination therapies are commonly used, clinical evidence demonstrating the beneficial effects of this treatment approach is lacking^46^, with tuberculosis treatment being a notable exception.^3^^,^^47^ Some increase in clinical efficacy has been observed on combinations that exhibit synergistic interactions *in vitro.*^2^^,^^46^ However, a clear correlation between antibiotic synergy and clinical improvement is yet to be established. Some studies link antibiotic synergy to better clinical outcome ^48^^,^^49^ whereas others do not.^46^^,^^50^, ^51^, ^52^

A key contributing factor to this discrepancy is that contrary to monotherapy schemes, combination treatments are prescribed empirically. As soon as antibiotic susceptibility data become available, treatment is usually refined (typically to monotherapy) or switched.^7^^,^^12^ That initial treatment however is prescribed both without any prior *in vitro* knowledge of antibiotic interaction profiles and with a lack of explicit clinical guidelines.

Data driven guidelines for combination therapy could bridge the gap between *in vitro* diagnostics and clinical outcome. However, systematizing interactions testing to that scale would require suitable clinical tests that quantify antibiotic interactions. Such tests should allow for the same systematic case-by-case-testing performed for monotherapy schemes.

For the specific case of β-lactam + aminoglycoside combination there are conflicting reports on whether β-lactam monotherapy is better or worse than the combination^52^, ^53^, ^54^. In light of the lack of evidence driven guidelines, we set out to perform an extensive quantification of antibiotic interactions in clinical strains. In BSI *E. coli,* we found that interaction profiles of the same antibiotic combinations varied significantly across strains, with responses to combinations ranging from synergistic additivity to substantial antagonism. This finding, also illustrated by different analyses and meta analyses of clinical data, as well as *in vitro* studies^46^^,^^52^^,^^53^^,^^55^, is characteristic of β-lactam and aminoglycoside combinations. Without prior knowledge of such variable *in vitro* responses to antibiotic combinations, antagonistic interactions would remain undetected. Approximately 3% of our screened strains exhibited clinically antagonistic interactions. That is a potential risk with this specific combination choice as it could potentially reduce treatment efficacy and be a contributing factor to the underperformance of clinical combination treatments. It should also be noted that of the 4 combinations tested, none had a significant synergistic effect against any of the 254 strains.

In empirical antibiotic combination treatments, antibiotics of the same class are often used interchangeably according to regional preferences.^34^^,^^54^ However, this study demonstrates that antibiotics from the same class, when combined with others, were not necessarily interchangeable. The interaction profile of a strain in the combination of AMP with GEN was highly correlated with its profile in the combination of CTX with GEN. Similarly, the profile of a strain for AMP with TOB was correlated with its profile in CTX with TOB. Taking these cross correlations into account suggests that the choice of aminoglycoside in the combination is what determines the interaction profile for these two drug combinations in this set of *E. coli* blood stream infection strains.

In this study, we focused on strains that are susceptible to both antibiotic classes for two reasons. Firstly, in a clinical setting, after appropriate susceptibility testing, treatment is modified to exclude antibiotics to which a strain is resistant to. Secondly, single or double resistant strains would reach levels of clinical resistance to antibiotics with a multitude of different resistance mechanisms and mutations.^56^, ^57^, ^58^, ^59^ The effect different resistance mechanisms will have on the interaction profile of the combinations could arguably be mechanism specific. Therefore, highly resistant strains, without further examination other than susceptibility, would not represent a homogenous population in which population-wide trends can be examined. However, with antibiotic resistance on the rise, drug combinations represent attractive therapy schemes especially in resistant and multi-resistant strains.^3^^,^^5^^,^^10^^,^^60^ Although outside the scope of this work, we believe that a systematized quantification of the interaction profiles of different combinations using large collections of clinical isolates to identify within-species variability could be beneficial in the way we treat multi-resistant infections. An isolate-stratified examination of how different mechanisms of resistance effect the interaction profiles of common clinical drug combinations represents a logical next step of this study, as it can be crucial in understanding and systematizing empirical use of drug combinations in the clinic.

Taking the findings of this study into account is a discouraging indication of the suitability of this empirical treatment for BSI *E. coli.* Especially since of the multiple β-lactam-aminoglycoside combinations tested here, not one exhibited a synergistic interaction. However, in multiple cases the combinations had antagonistic and even suppressive effects. The detection of antagonistic interactions between drugs used in treatment for sepsis raises a valid concern on this clinical practice, and it motivates the need for strain-specific interaction testing to avoid antibiotic combinations that might reduce the efficacy of treatment Having data driven recommendations for the use of *in vitro* testing of antibiotic combinations could prove to be beneficial clinical practice. Other combination schemes might be identified to show less risk and perhaps exhibit *in vitro* synergy. Finally, these findings also reveal a potential clinical trade-off that needs to be considered between the beneficial effect of increased coverage of antibiotic combinations versus the risk of antibiotic antagonism that can reduce treatment efficiency.

## Declaration of interests

Dan I. Andersson and Nikos Fatsis-Kavalopoulos are involved in patent application SE 2050304-1 relating to the CombiANT method. Remaining author has no declaration of interest to disclose.

## Appendix Supplementary materials

Image, image 1

Image, image 2

Image, application 1

Image, application 2
