# Supplementary figures and images for: Potential risks of treating bacterial infections with a combination of β-lactam and aminoglycoside antibiotics: A systematic quantification of antibiotic interactions in E. coli blood stream infection isolates
Source: eBioMedicine. 2022 Apr 1;78:103979. doi: 10.1016/j.ebiom.2022.103979 (PMC8983351; doi:10.1016/j.ebiom.2022.103979)

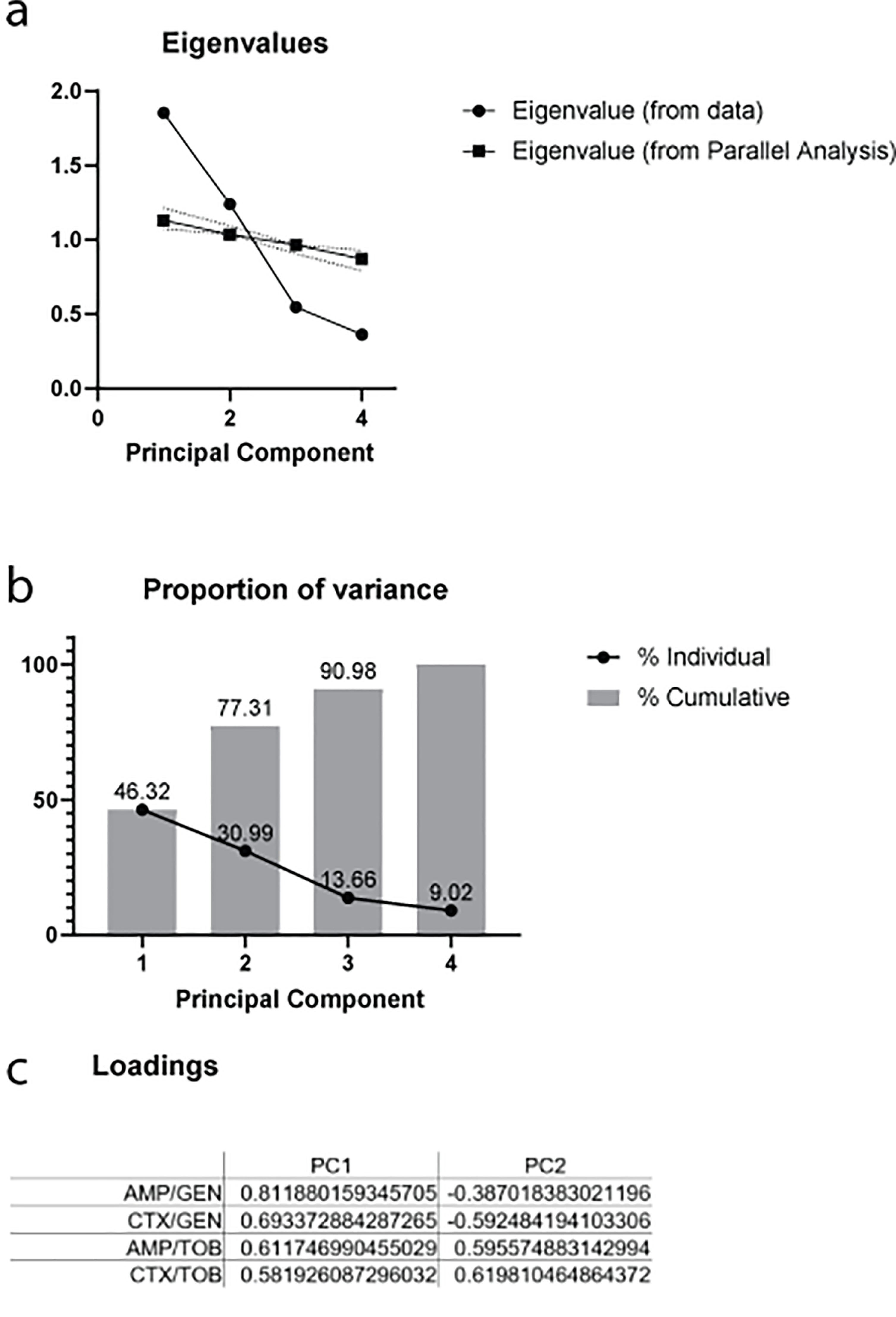

Supplement: Supplementary file 1 [file mmc1.jpg]

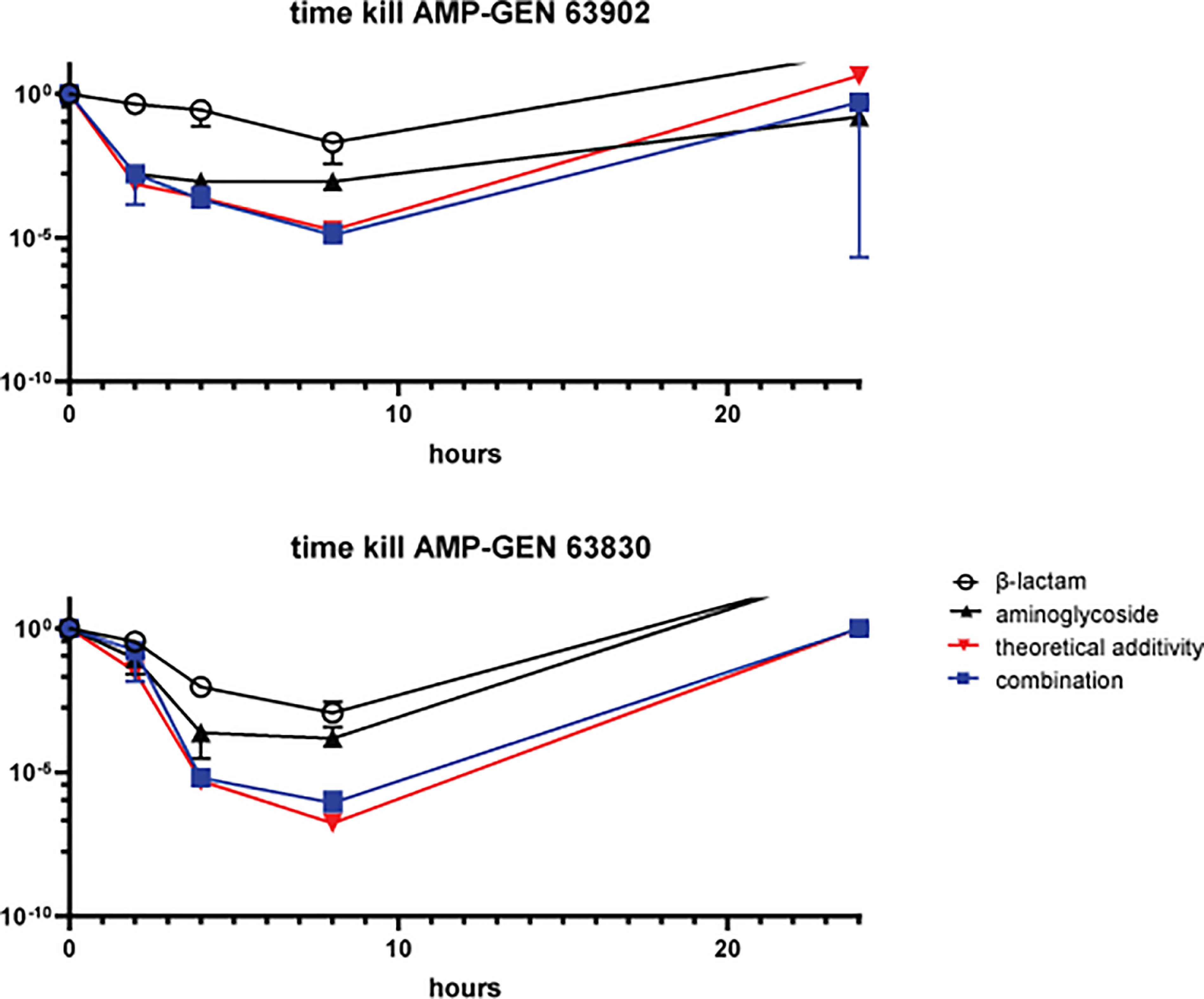

Supplement: Supplementary file 2 [file mmc2.jpg]
