# Supplementary material for: Potential risks of treating bacterial infections with a combination of β-lactam and aminoglycoside antibiotics: A systematic quantification of antibiotic interactions in E. coli blood stream infection isolates
Source: eBioMedicine. 2022 Apr 1;78:103979. doi: 10.1016/j.ebiom.2022.103979 (PMC8983351; doi:10.1016/j.ebiom.2022.103979)
Supplement: Supplementary file 4 [file mmc4.docx]

|  |  |  |  |  |  |  |
| --- | --- | --- | --- | --- | --- | --- |
|  | 65,6% of the 500 strains tested were susceptible to all antibiotics | | | | |  |
|  | 44,4% of the 500 strains tested showed resistance to at least 1 antibiotic | | | | | |
| 7.48792271 | 7,5% of the population showed resistance to 2 of the antibiotics | | | | |  |
| 3.62318841 | 3,6% of the population showed resistance to 3 or more antibiotics | | | | |  |
|  |  |  |  |  |  |  |
|  | Within the resistant strains: | | |  |  |  |
|  | 66.1764706 |  | 66% showed resistance to at least 1 antibiotic | | | |
|  | 22.7941176 |  | 23% showed resitance to two of the antibiotics | | | |
|  | 11.0294118 |  | 11% showed resistance to 3 or more antibiotics | | | |
|  |  |  |  |  |  |  |
